# Campbell Title Registrations to Date – July 2020

**DOI:** 10.1002/cl2.1108

**Published:** 2020-09-02

Details of new titles for systematic reviews or evidence and gap maps that have been accepted by the Editor of a Campbell Coordinating Group are published in each issue of the journal. If you would like to receive a copy of the approved title registration form, please send an email to the Managing Editor of the relevant Coordinating Group.

## 1 CRIME AND JUSTICE

*The use of media as an offence‐related behaviour in recidivism of ex‐offenders: A systematic review*

Ian S. Hamilton, Dominic A. S. Pearson

18 June 2020

*Online Interventions for reducing hate speech and radicalization: A systematic review*

Steven Windisch, Ajima Olaghere, Susann Wiedlitzka

28 May 2020

*Economic, health, and safety impacts of marijuana liberalization laws: An evidence and gap map*

Eric L. Sevigny, Rosalie L. Pacula, Ariel M. Aloe, Danye N. Medhin, Jared Greathouse

27 May 2020

## 2 DISABILITY

*Support interventions for children with intellectual disabilities: An evidence and gap map*

Huijuan Li, Xiuxia Li, Meixuan Li, Liang Yao, Kehu Yang

27 April 2020

## 3 EDUCATION

*High school esports: Describing the current state of the literature and exploring effects on student outcomes*

Brandy Maynard, Jacob A. Maynard

30 June 2020

## 4 INTERNATIONAL DEVELOPMENT

*The impact of COVID‐19 on the health and nutrition of women and children in low‐ and middle‐income countries*

Bianca Carducci, Emily C Keats, Anushka Ataullahjan, Zulfiqar A. Bhutta

9 July 2020

## 5 METHODS

*Searching and reporting in Campbell Collaboration systematic reviews: An assessment of current methods*

Sarah Young, Alison Bethel, Ciara Keenan, Kate Ghezzi‐Kopel, Elizabeth Moreton, David Pickup, Zahra Premji, Morwenna Rogers, Jinhui Tian, Bjørn Christian Arleth Viinholt, Xiaoqin Wang

29 June 2020

*Machine learning methods for moderator meta‐analysis: A systematic meta‐review and an application to tutoring interventions*

Rasmus Henriksen Klokker, Jens Dietrichson, Trine Filges, Terri D. Pigott, Bjørn Arleth Viinholt

8 June 2020

*Frameworks for stakeholder engagement in guideline development: a systematic review*

Jennifer Petkovic, Alison Riddle, Elie Akl, Joanne Khabsa, Lyubov Lytvyn, Vivian Welch, Thomas Concannon, Peter Tugwell

16 April 2019

*Assessing the impact of stakeholder engagement in guideline development: a mixed‐methods systematic review*

Joanne Khabsa, Lyubov Lytvyn, Alison Y. Riddle, Jennifer Petkovic, Elie Akl, Thomas Concannon, Peter Tugwell

16 April 2019

*Barriers and facilitators to stakeholder engagement in guideline development: a mixed‐methods systematic review*

Alison Y. Riddle, Jennifer Petkovic, Elie Akl, Joanne Khabsa, Lyubov Lytvyn, Vivian Welch, Thomas Concannon, Peter Tugwell

16 April 2019

## 6 SOCIAL WELFARE

*Gender, mental health, and family violence: The indirect impacts of COVID‐19 on women and children in low‐ and middle‐income countries*

Anushka Ataullahjan, Bianca Carducci, Emily Keats, Zulfiqar A. Bhutta

24 June 2020

*Community‐based interventions for initiating early end‐of‐life conversations in non‐terminally ill adults: A systematic review*

Mervyn Lim, Zachus Tan, Joel Foo, Noreen Chan, Huso Yi, Miny Samuel

16 June 2020

*Occupational health and safety policy and regulatory enforcement to improve work environment at workplaces: An evidence and gap map*

Anja Bondebjerg, Trine Filges, Jan Hyld Pejtersen, Bjørn Christian Arleth Viinholt

3 June 2020

*What are the individual psychological factors that predict a positive adjustment to retirement?*

Denise Taylor, Rachel Lewis, Joanna Yarker

2 June 2020

*Effectiveness of social accountability interventions in Asia: An evidence and gap map*

Mirza Hassan, Howard White, Ashrita Saran, Maria Matin, Iffat Zahan, Shamael Ahmed

27 May 2020

*Effectiveness of psychological and psychosocial rehabilitation interventions in child sexual abuse survivors: A systematic review*

Kunjan Kunjan, Meenakshi Sharma, Nidhi Chauhan, Anil Thota

25 May 2020

*Evidence and gap map of the effects of health interventions on out‐of‐pocket expenses*

Xiuxia Li, Yanfei Li, Rui Li, Nan Chen, Kangle Guo, Jieyun Li, Huijuan Li, Meixuan Li, Howard White, Kehu Yang

24 April 2020

